# Supplementary material for: Dyshomeostatic modulation of Ca2+-activated K+ channels in a human neuronal model of KCNQ2 encephalopathy
Source: eLife. 2021 Feb 5;10:e64434. doi: 10.7554/eLife.64434 (PMC7864629; doi:10.7554/eLife.64434)
Supplement: Transparent reporting form [file elife-64434-transrepform.docx]

***eLife’s* transparent reporting form**

We encourage authors to provide detailed information *within their submission* to facilitate the interpretation and replication of experiments. Authors can upload supporting documentation to indicate the use of appropriate reporting guidelines for health-related research (see [EQUATOR Network](http://www.equator-network.org/%20)), life science research (see the [BioSharing Information Resource](https://biosharing.org/)), or the [ARRIVE guidelines](http://www.plosbiology.org/article/info:doi/10.1371/journal.pbio.1000412) for reporting work involving animal research. Where applicable, authors should refer to any relevant reporting standards documents in this form.

If you have any questions, please consult our Journal Policies and/or contact us: [editorial@elifesciences.org](mailto:editorial@elifesciences.org).

**Sample-size estimation**

- You should state whether an appropriate sample size was computed when the study was being designed
- You should state the statistical method of sample size computation and any required assumptions
- If no explicit power analysis was used, you should describe how you decided what sample (replicate) size (number) to use

All statistical methods and sample sizes are included in the Methods, Results and Figures and Figure Legends. An appropriate sample size could not be estimated before experiments because we did not have any prior knowledge about effects of our manipulations. Our requirement was to have at least 2 independent biological replicates for each type of experiment (MEA, current-clamp and qPCR) as well as at least two independent technical replicates per assay.

Please outline where this information can be found within the submission (e.g., sections or figure legends), or explain why this information doesn’t apply to your submission:

**Replicates**

- You should report how often each experiment was performed
- You should include a definition of biological versus technical replication
- The data obtained should be provided and sufficient information should be provided to indicate the number of independent biological and/or technical replicates
- If you encountered any outliers, you should describe how these were handled
- Criteria for exclusion/inclusion of data should be clearly stated
- High-throughput sequence data should be uploaded before submission, with a private link for reviewers provided (these are available from both GEO and ArrayExpress)

Please outline where this information can be found within the submission (e.g., sections or figure legends), or explain why this information doesn’t apply to your submission:

Biological replicates were defined as independent differentiations of iPSCs into cortical neurons. Technical replicates were defined as: wells on MEA plates, neurons patched in whole-cell current clamp experiments or cDNA sample/primer pair replicates in qPCR experiments. All of this information is within each figure legend for each figure. With the exception of MEA and qPCR data in figure 6 (F-G) and MEA data in Figure 5 (D) all data comes from 3 or more independent biological replicates. Outliers are defined for MEA and patch data in the methods sections respectively. We did not have any outliers for qPCR data. WGS data has the potential to personally identify the deidentified donor, thus we are not able to disclose it at this time.

**Statistical reporting**

- Statistical analysis methods should be described and justified
- Raw data should be presented in figures whenever informative to do so (typically when N per group is less than 10)
- For each experiment, you should identify the statistical tests used, exact values of N, definitions of center, methods of multiple test correction, and dispersion and precision measures (e.g., mean, median, SD, SEM, confidence intervals; and, for the major substantive results, a measure of effect size (e.g., Pearson's r, Cohen's d)
- Report exact p-values wherever possible alongside the summary statistics and 95% confidence intervals. These should be reported for all key questions and not only when the p-value is less than 0.05.

Please outline where this information can be found within the submission (e.g., sections or figure legends), or explain why this information doesn’t apply to your submission:

All statistical information including statistical test and post hoc test, exact Ns, mean ± SEM, exact p-values (even if p > 0.05) and degrees of freedom F value (for two-way or repeated measure ANOVA) is presented in each figure legend for every figure and in tables 1 and 2.

(For large datasets, or papers with a very large number of statistical tests, you may upload a single table file with tests, Ns, etc., with reference to sections in the manuscript.)

**Group allocation**

- Indicate how samples were allocated into experimental groups (in the case of clinical studies, please specify allocation to treatment method); if randomization was used, please also state if restricted randomization was applied
- Indicate if masking was used during group allocation, data collection and/or data analysis

Please outline where this information can be found within the submission (e.g., sections or figure legends), or explain why this information doesn’t apply to your submission:

We compared neurons differentiated from a patient-derived iPSC line to the same line which had the patient (*KCNQ2*) mutation corrected using CRISPR/Cas9 gene editing. This information is found in the methods section.

**Additional data files (“source data”)**

- We encourage you to upload relevant additional data files, such as numerical data that are represented as a graph in a figure, or as a summary table
- Where provided, these should be in the most useful format, and they can be uploaded as “Source data” files linked to a main figure or table
- Include model definition files including the full list of parameters used
- Include code used for data analysis (e.g., R, MatLab)
- Avoid stating that data files are “available upon request”

Please indicate the figures or tables for which source data files have been provided:

Source data files have been provided for Figures 1, 2, 3, 4, 5, 6 and their figure supplements.

All code is available for download at github.com/simkind/Patch-clamp-analysis.git
